# Parent-administered Metered-dose Inhalers Improves Medication Administration Time in the Children’s Emergency

**DOI:** 10.1097/pq9.0000000000000889

**Published:** 2026-07-20

**Authors:** Nur Nasiha B.M. Bahrudin, Cherie Tan, Germaine R. Eu, Qian Wen Sng

**Affiliations:** From the * Department of Emergency Medicine, KK Women’s and Children’s Hospital, Singapore; † Nursing Clinical Services, KK Women’s and Children’s Hospital, Singapore.

## Abstract

**Introduction:**

Prompt administration of bronchodilator therapy via metered-dose inhalers (MDIs) is critical to optimizing clinical outcomes in children with bronchospasm. At our children’s emergency department, baseline data indicated that the average interval between the first and second MDI bronchodilator cycles was 44.4 minutes, highlighting delays in bronchodilator therapy. The primary objective was to achieve a 30% reduction in the time interval between the first and second bronchodilator MDI cycles within 1 year.

**Methods:**

This multidisciplinary quality improvement project was conducted using the Plan-Do-Study-Act model. Children aged 4–13 years presenting with clinical symptoms requiring bronchodilator therapy via MDI (salbutamol ± ipratropium) were included. Key interventions included revising the workflow to allow nurse-supervised parent administration of MDI, supported by information leaflets, educational videos, nurse-led demonstrations, competency checks, and a designated space for MDI administration. Our primary outcome measure was the time taken to administer the second cycle. Secondary outcome measures focused on parental competency and parent and nurse satisfaction.

**Results:**

From August 2022 to June 2023, our educational program reduced the mean time to administer the second MDI cycle from 44.3 (SD 31.3) to 27.6 (SD 8.1) minutes (*P* < 0.001). Ninety-eight percent of parents were competent on their first attempt. Both parents and nurses reported high satisfaction with the initiative and perceived it as safe.

**Conclusions:**

Parent-administered MDI significantly reduced the time interval between the first and second cycles and was highly accepted by parents and nurses, demonstrating an effective, sustainable approach to reducing delays in MDI administration in the emergency department.

## INTRODUCTION

Prompt administration of inhaled bronchodilators is essential for patients who require bronchodilator therapy.^1^ According to the Global Initiative for Asthma, the use of inhaled beta-agonists (salbutamol) and anticholinergics (ipratropium) delivered by metered-dose inhaler (MDI) with spacers every 20 minutes in the first hour is the primary treatment for acute asthma.^2,3^ This approach provides rapid bronchodilation, improving airflow and symptoms such as dyspnea and wheeze.^4^ At our institution, these guidelines are adapted to initiate treatment for both asthmatic and nonasthmatic patients presenting with physician-assessed bronchospasm/wheeze. Previous improvement project (QI) projects have focused on improving the timeliness of short-acting β-agonists and corticosteroid administration by implementing interventions such as standardized treatment pathways and nurse-initiated protocols. ^5–8^

At our institution, delayed bronchodilator MDI administration was identified as an area for improvement after baseline data showed an average delay of 44.4 minutes between the first and second MDI cycles. This delay significantly exceeded our heuristic target of 20 minutes, which is considered optimal for a therapeutic response.

### QI Framework and Rationale

**Fig. 1.**
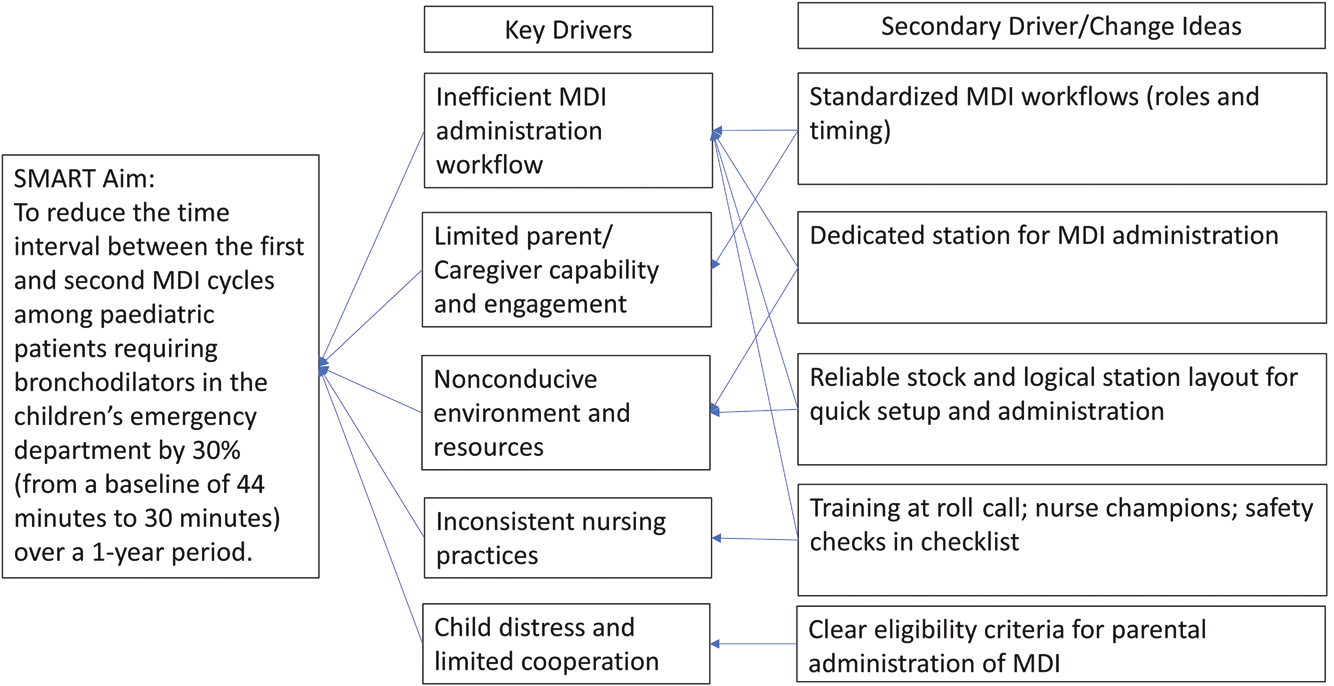
Key driver diagram depicting the project aim statement, key drivers, and change ideas.

Guided by the Model of Improvement framework,^9^ an interdisciplinary QI team consisting of a pediatric emergency medicine physician, nurse clinicians from the emergency and intensive care unit, emergency department (ED) staff nurses, and 2 hospital QI analysts was assembled. The team collaboratively reviewed existing workflow processes and baseline data to identify factors contributing to delays in MDI administration. A fishbone diagram and a key drivers diagram (Fig. 1) were developed to identify potential root causes and guide the design of targeted interventions. (**See Supplemental Digital Content 1**, which displays the fishbone diagram, https://links.lww.com/PQ9/A763.) The primary drivers identified were (1) inefficient MDI administration workflow, (2) limited parent capability and engagement, (3) nonconducive environment and resources, (4) inconsistent nursing practices, and (5) child distress and limited cooperation.

## SPECIFIC AIM

The QI project aimed to enhance the timeliness and efficiency of bronchodilator MDI administration in the ED by introducing a parental MDI administration workflow supported by structured educational resources. The specific primary objective was to achieve a 30% reduction in the time interval between the first and second MDI cycles within 1 year. The secondary objectives were to achieve a parental MDI administration competency rate of at least 80%, positive ratings for satisfaction and perceived safety among at least 80% of parents, and positive ratings for nurse satisfaction and perceived safety among at least 80% of nurses.

## METHODS

### Context

We conducted the QI study at KK Women’s and Children’s Hospital from August 2022 to June 2023. The ED is the largest tertiary children’s hospital in Singapore, with an annual attendance of approximately 142,000 children. Among these, respiratory conditions such as asthma and bronchitis are the most common emergent diagnoses.^10^ The designated area within the ED, “Observation 1,” primarily caters to children requiring MDI therapy and short-term observation, with a dedicated corner equipped for MDI administration and monitoring. This area is overseen by a team of 3–4 nurses per shift, each proficient in MDI administration.

### Inclusion Criteria

The QI initiative focused on children aged 4–13 years who presented with clinician-assessed bronchospasm/wheeze and required bronchodilator therapy via MDI (salbutamol ± ipratropium). We included patients presenting with category P2+ on the 4-level Singapore Pediatric Triage Scale^11^ who required at least 2 bronchodilator MDI cycles. (**See Supplemental Digital Content 2**, which displays the Singapore Pediatric Triage Scale, https://links.lww.com/PQ9/A764.) Children who required supplemental oxygen were classified as category P1, and participants who were unwilling to participate were excluded. Families meeting the above criteria were identified and recruited in the ED during the nurse’s assessment. The same eligibility criteria were applied to retrospectively identify the baseline cohort.

### Interventions

Our interventions were implemented using 2 Plan-Do-Study-Act (PDSA) cycles: (1) a workflow shift to a nurse-supervised, parent-administered second-dose bronchodilator MDI therapy, and (2) creation of a dedicated station for effective MDI administration. During the implementation period, the team met twice a week to review the workflow and interventions, incorporating feedback from the QI team and nurses. The project’s workflow and resources were disseminated to all nurses during roll call sessions to ensure standardized data collection procedures.

#### PDSA 1: Nurse-supervised Parent Administration of MDI Bronchodilators

**Fig. 2.**
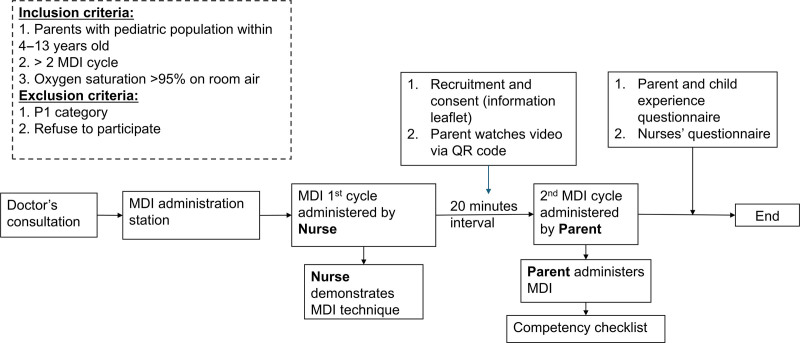
MDI administration intervention workflow.

As part of the initial interventions in August 2022, we introduced a structured MDI intervention workflow (Fig. 2), a parental MDI competency checklist, and an information leaflet that included a link to an educational video on MDI administration. (**See Supplemental Digital Content 3**, which displays the MDI competency checklist, https://links.lww.com/PQ9/A765.) (**See Supplemental Digital Content 4**, which displays the parent self-administered MDI program information leaflet, **https://links.lww.com/PQ9/A766**.) Children who were assessed as requiring MDI bronchodilator therapy were guided to the administration station at Observation 1 by their attending physician, where the nurse would administer the first MDI cycle. Before medication administration, a set of vital signs, specifically heart rate, respiratory rate, and oxygen saturation levels, was assessed and charted in the electronic medical record (EMR) (Sunrise Clinical Manager). MDI therapy was administered via a spacer chamber and an appropriately sized mask.

Although administering the first cycle, the nurse concurrently explained the steps of proper MDI-spacer technique to ensure prompt medication administration. Upon completion, the nurse marked the medication order for the first MDI administration time as complete. Throughout this process, the nurse assessed the child’s suitability and tolerance to participate in the study. If deemed cooperative, the project was introduced to the child’s parent. Following verbal consent to participate, the nurse provided the parents with the information leaflet outlining the program’s objectives, anticipated outcomes, and interventions. Parents were encouraged to seek clarification on any aspects of the administration process and technique. The leaflet included a QR code that links to a comprehensive, 5-minute, step-by-step instructional video on “Parent-administered MDI.” The nurse instructed the parent to return in 20 minutes for the next dose. Although waiting, parents would watch the instructional video to reinforce proper techniques.

During the second MDI cycle, an ED nurse assessed parents’ MDI administration technique using an 11-step competency checklist (**Supplemental Digital Content 3**, https://links.lww.com/PQ9/A765). Parents were deemed competent if they satisfactorily completed all the steps on the checklist. If mistakes occurred, the nurse intervened to correct the parent. After administration, the nurse marked the medication order as complete in the EMR, indicating the time of the second MDI administration.

Additionally, the nurse provided feedback and comments based on the parent’s performance. Parents assessed as competent administered subsequent bronchodilator MDI therapy with minimal guidance and supervision. This approach improves efficiency by allowing nurses to attend to other clinical tasks, such as documenting the child’s vital signs, preparing any other prescribed oral medications, and attending to other children requiring MDI administration. After the prescribed MDI cycles, parents completed the 10-item Parent and Child Experience Survey (Table 2) before the postmedication review. The nurse also completed a 5-item questionnaire (Table 3) after the intervention.

#### PDSA 2: Designating a Conducive Space for MDI Administration

In December 2022, we gathered to review the revised workflow and feedback from ED staff nurses. Nurses faced challenges in the current MDI administration area, including overcrowding and frequent distractions from patients and caregivers. In March 2023, the team acquired a consultation room to accommodate patients who required nursing care but *did not* require MDI therapy. By redistributing patients, we created a more conducive environment in “Observation 1” for nurses to focus solely on MDI administration and parental education.

### Measures

The primary outcome measure was the interval between the first and second MDI cycles. Baseline outcome data were retrospectively abstracted from the EMR (January 2022–July 2022). During the intervention period (August 2022–June 2023), the team measured the same outcomes and reviewed them twice a month. Data collection continued during the sustainability phase (May 2024–January 2025) to monitor ongoing improvements.

The secondary outcomes of the study focused on evaluating parental competency in independently administering MDI to their children, the parent and child experiences, and nurse satisfaction with the initiative. Parental competency in MDI administration was assessed by nurses using the 11-step competency checklist, which was adapted from our department’s competency guidelines and established standards (**Supplemental Digital Content 3**, https://links.lww.com/PQ9/A765). The Parent and Child Experience Survey, adapted from Osmanlliu et al,^12^ included 10 items in total (Table 2). Parental experience encompassed key areas, including perceived program benefits, the safety of the care environment, the quality of nurse supervision, and parents’ willingness to participate in the program again. It also evaluated overall satisfaction with the program, improvements in parents’ understanding of proper MDI administration techniques, and their perceptions of their child’s level of anxiety. The child’s experience was assessed through self-report for children aged 7 and older, focusing on their comfort and emotional response to receiving MDI from either the parent or the nurse.

Additionally, nurses were tasked with completing a 5-item questionnaire that evaluates their satisfaction with the program, specifically whether the program was beneficial, its impact on workload, perceived safety, overall satisfaction, and their willingness to teach parents MDI administration in the future. Nurses also provided feedback on any implementation issues through an open-ended question.

### Analysis

Data cycles were analyzed using an X-bar chart. Statistical process control charts were generated using QI Charts (v2.0.23, Scoville Associates, 2009) for Microsoft Excel. We applied special-cause rules and shifted the mean after 8 or more consecutive points on 1 side of the centerline before recalculating limits for the subsequent segment.^13^ Descriptive statistics were used to summarize children’s sociodemographic and clinical characteristics and parents’ and nurses’ satisfaction surveys. Independent 2-sample *t* tests were performed to identify significant differences in interval duration between the first and second MDI cycles during the baseline and intervention periods. Statistical analysis was performed using Stata,^14^ and statistical significance was set at a *P* value of less than 0.05. This article was prepared in accordance with the SQUIRE 2.0 reporting guideline.^15^

### Ethical Considerations

The study was approved by the SingHealth Centralized Institutional Review Board (CRIB 2022/2499).

## RESULTS

During the baseline period (January 2022–July 2022), 110 patients were identified for inclusion in the study (Table 1). From August 2022 to June 2023, 100 eligible patients participated in the study. However, data on MDI administration duration were available for only 85 participants in the postimplementation group due to missing data and technical issues with the electronic health record system. The mean age was 6.1 years (SD 2.1 y). Fifty-four percent of children presented with tachypnea (n = 47), 66% with wheezing (n = 57), 84% with shortness of breath (n = 73), and 60% had cough (n = 52), all of which required MDI therapy.

**Table 1.** Patient Characteristics and Demographics in the Baseline (n = 110) and Intervention (n = 85) Period

	Baseline (n = 110)	Intervention (n = 85)	*P*
Age, y, mean (SD)	6.1 (2.1)	6.5 (2.8)	0.288^*^
PACS, n (%)			
P1	0	1 (1.2)	
P2	0	6 (7.1)	0.013^†^
P2+	110 (100.0)	77 (90.6)	
P3	0	1 (1.2)	
Main diagnosis description, n (%)			
Acute bronchitis/bronchitis	63 (57.3)	42 (49.4)	0.269^†^
Asthma and bronchial hyperreactivity	39 (35.5)	32 (37.6)	
Lower respiratory tract conditions	6 (5.5)	7 (8.2)	
Upper respiratory tract conditions	2 (1.8)	1 (1.2)	
Other isolated diagnoses	0 (0)	3 (3.5)	

* Independent *t* tests.

† Chi-square tests.

PACS, Patient Acuity Category Scale.

### Primary Outcome

**Fig. 3.**
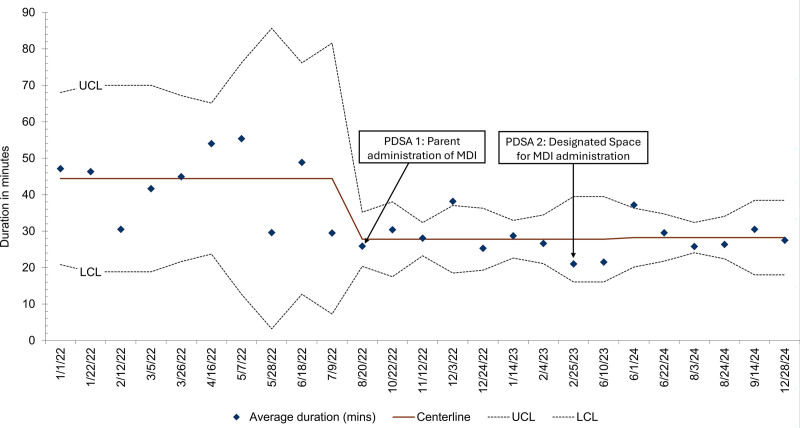
X-bar chart: average duration between first and second MDI doses (min). LCL, lower control limit; UCL, upper control limit.

The average baseline interval between the first and second MDI administrations was 44.4 minutes (SD 31.3 min). Following the introduction of the workflow for parental administration of MDI (PDSA cycle 1), we observed a centerline change that lowered the average time interval between the first and second MDI by 37.8% to 27.6 minutes (SD 8.1 min) (*P* < 0.01) (Fig. 3). Moreover, 8 children received their second MDI within 20 minutes, meeting our heuristic goal. We did not observe additional improvements with the designation of “Observation 1” (PDSA 2). Data collected after the intervention (May 2024–January 2025) remained within the recalculated control limits. They showed no evidence of special-cause variation, confirming the stability of the reductions in MDI administration duration (n = 57, mean = 28.4, SD = 7.3).

### Secondary Outcome

#### Parental Competency in MDI Administration

The majority of participating parents (n = 83, 98%) were competent in independently administering MDI to their children upon program completion, with 2 (2%) deemed “incompetent” on their first assessment but subsequently assessed as competent on their second assessment.

#### Parental Satisfaction in MDI Administration

Most parents were satisfied or very satisfied with the program (n = 99, 99%) and perceived it as safe or very safe (n = 99, 99%). All participating parents (n = 100, 100%) reported perceiving this program as beneficial to them, with “increased knowledge on inhaler administration technique” (n = 78, 78%), “my child’s comfort” (n = 73, 73%), and “opportunity to learn about my child’s condition” (n = 67, 67%) identified as the top 3 perceived benefits. Most felt that the nurses’ supervision was sufficient or excellent (n = 99, 99%). Despite this, only 76 (76%) reported being keen to participate in the program again, and half felt that the interventions did not alleviate their child’s anxiety (n = 54, 55.1%). Most children (n = 44, 68.8%) preferred their parents to administer the inhaler, but reported “did not feel scared” when the nurse administered the MDI (n = 57, 89.1%) (Table 2).

**Table 2.** Parent (n = 100) and Child (n = 64) Experience With the Parent-administered MDI Initiative

Questions	Results, n (%)
Parent satisfaction	
Did you find this program beneficial to you?	
Yes	100 (100)
If you have chosen yes, what were the benefits of this program?	
My child’s comfort	73 (73)
Opportunity to learn about my child’s condition	67 (67)
Opportunity to be supervised during treatments	64 (64)
A safe care environment	46 (46)
Parental empowerment or involvement	65 (65)
It has increased my knowledge of inhaler administration technique	78 (78)
Is this a safe initiation for your child in a CE setting?	
Very safe	53 (53)
Safe	46 (46)
Unsafe	0 (0)
Very unsafe	0 (0)
How did you find the level of the nurses’ supervision?	
Insufficient	1 (1)
Sufficient	20 (20)
Excellent	79 (79)
How will you rate your overall level of satisfaction with this program?	
Very satisfied	54 (54)
Satisfied	45 (45)
Unsatisfied	1 (1)
Very unsatisfied	0 (0)
If your child were to return to CE for a similar reason, would you want to participate in this program again?	
Yes	76 (76)
How has this program affected your child’s level of anxiety?	
My child was less anxious	54 (55.1)
My child was more anxious	8 (8.2)
It made no difference	36 (36.7)
Knowledge of MDI	
Has this program made an impact on your level of understanding of MDI administration technique and education?	
Yes	84 (92.3)
No	7 (7.7)
It made no difference	0 (0)
Child experience	
Do you prefer the nurse or your parents to give you the inhaler?	
Parent	44 (68.8)
Nurse	20 (31.2)
How do you feel when the nurse gives you the inhaler?	
I feel scared	7 (10.9)
I do not feel scared	57 (89.1)

CE, children’s emergency.

#### Nurses’ Satisfaction

All the nurses had positive experiences with the program, rating it as satisfied or very satisfied (n = 100, 100%), and most perceived it as safe or very safe (n = 99, 99%) (Table 3). A significant proportion of nurses (n = 99, 99%) acknowledged tangible benefits of the program. Specifically, 88% (n = 88) affirmed its effectiveness in reducing their workload, whereas 59% (n = 59) felt that it facilitated their ability to concentrate on other clinical tasks.

**Table 3.** Nurses’ (n = 100) experience with the parent-administered MDI initiative

Questions	Results, n (%)
Do you think that this program is beneficial to you? n (%)	
Yes	99 (99)
If yes, how has this program benefited you?	
It reduces the nurses’ workload	88 (88)
I am able to concentrate on other clinical tasks	59 (59)
Less distraction	24 (24)
How do you perceive the safety of this program?	
Very safe	55 (55)
Safe	44 (44)
Unsafe	1 (1)
Very unsafe	0 (0)
Will you be keen to teach parents how to administer MDI for their child in the future?	
Strongly agree	78 (78)
Agree	21 (21)
Neutral	1 (1)
Disagree	0 (0)
Strongly disagree	0 (0)
How would you rate the overall satisfaction of this program?	
Very satisfied	84 (84)
Satisfied	16 (16)
Unsatisfied	0 (0)
Very unsatisfied	0 (0)

## DISCUSSION

This QI initiative introduced a standardized workflow for nurse-supervised parent-administered MDI in the ED. In alignment with our primary goal, implementation of the initiatives resulted in a 37.8% reduction in the time between the first and second MDI cycles, reducing the mean time to 27.6 minutes. We surpassed our target of a 30% improvement and sustained the result for 9 months. Furthermore, the initiatives achieved our secondary goals for assessed parental competency, parental experience, and nurses’ satisfaction.

### MDI Administration Workflow Redesign and Impact on Timeliness

Previous studies aimed at improving the timeliness of medication administration for patients with acute asthma have implemented a variety of strategies, including physician and nurse education, asthma clinical pathways, nurse-driven systems, and medication standardization.^7,16,17^ In contrast, we reduced the delays by enabling nurse-supervised parental administration of MDI within a standardized workflow. Two operational mechanisms improved efficiency: (1) shifting nurses’ role from end-to-end, one-to-one MDI administration to safety check and supervision, thereby freeing minutes of direct care time,^18^ and (2) using a dedicated MDI station that allows concurrent oversight of multiple families. Together, these changes increased concurrent nursing coverage for more families and throughput, mitigating the bottleneck at the 20-minute redosing point, as reflected in the substantial reduction and decreased variation in the second-dose interval. Our findings suggest that embedding supervised parental administration into the ED workflow may address healthcare professionals’ perceptions that MDI is time-consuming, thereby supporting sustained MDI use over nebulizers.^19,20^

### Parental Competency in MDI Administration

Despite frequent inhaler use, many parents still demonstrate poor MDI technique, which leads to ineffective disease management, increased admission frequency, and treatment expenses.^21,22^ Volerman et al^21^ and Benito-Ruiz et al^23^ emphasized the pivotal role of educators, such as physicians, advanced practice nurses, pharmacists, and nurses, in educating children and caregivers on MDI administration through various educational methods, including pamphlets, videos, and in-person demonstrations. Integrating multimedia interventions, such as instructional videos, is particularly effective for providing accessible, step-by-step guidance on inhaler use in both clinical settings and at home.^21^ Visits to the ED present an opportunity to identify errors in critical steps and address knowledge gaps among parents of children who require frequent inhaler use.^12^ With this in mind, we tailored our educational program to include a standardized checklist, a video guide, and a teach-to-goal method. A nurse-supervised parent-administered second MDI dose followed this education. In our project, 98% of parents achieved competency after instruction during the first MDI cycle. These results validated the use of demonstrations and face-to-face guidance as effective modalities for parents to educate and improve on MDI administration techniques in the ED.^23^

### Parents’ and Nurses’ Satisfaction

Given the intentional shift from nurse-only to nurse-supervised parent administration of MDI from the second cycle onward, we prespecified parent and nurse satisfaction and perceived safety as balancing measures to ensure that quality was not compromised. In our project, both parents and nurses expressed high levels of satisfaction and perceived the new workflow to be safe. Our findings were consistent with prior work showing high acceptance among parents of a nurse-supervised, parent-administered MDI in a pediatric ED.^12^ Caregiver involvement in medication administration is already supported in children’s wards, outpatient settings, and at home.^24–27^ Our project extends this established paradigm by embedding supervised parental MDI administration into the ED workflow. Nurses’ perceptions of the program’s benefits were more nuanced: although many nurses reported reduced workload (88%), only 59% agreed they could concentrate on other clinical tasks. This finding likely reflected ongoing responsibilities for medication and patient verification, supervision, and physiological monitoring in the current workflow.^22,28^

### Limitations

Although our project provides valuable insights, we acknowledge several limitations to consider when interpreting the findings. First, this single-department QI project used nonrandom sampling and had a small sample size, which could have introduced selection bias and limited the generalizability to settings with similar staffing models and policies regarding parental administration of medication. Second, our study’s focus on a specific age group, 4–13 years, may have limited its applicability to a wider age range of children who could tolerate it. Third, as nurses assessed most parents only once, or at most twice, this may be insufficient to determine whether the parent had fully achieved correct inhaler technique and could administer it independently. In fact, Kamps et al^29^ emphasized that at least 3 repeated instructional sessions were required to achieve correct inhaler technique among parents, especially first-time users. Additionally, variability among newly onboarded nurses may introduce inconsistencies in parent coaching and MDI administration techniques. Nonetheless, the workflow was overseen by nurses proficient in MDI administration, and resources were disseminated to all staff during roll calls. Finally, although the intervention demonstrated improvements by reducing delays in MDI administration, its broader impacts, such as time to disposition, hospitalization rate, and unplanned readmission rate, were not evaluated.

### Sustainability

To support the sustainability of the initiatives, we will embed the QI workflow and materials into the standard MDI administration workflow and incorporate periodic refresher training for all department nurses.

## CONCLUDING SUMMARY

Our project reduced the mean time between the first and second bronchodilator MDI doses by implementing a nurse-supervised, parent-administered workflow supported by an educational video, an 11-step competency checklist, and a designated MDI station. High parent and nurse satisfaction, along with perceived safety, further support implementation. The approach is low-cost and scalable to pediatric EDs that permit nurse-supervised parental administration of MDI. Future studies should expand to evaluate MDI self-administration among older children (ages 8–14 y) using a Teach-to-Goal approach to build autonomy while maintaining timely dosing.^21^

## ACKNOWLEDGMENTS

The authors thank the children’s emergency nurses at KK Women’s and Children’s Hospital, the MDI QI team (Head of Department Associate Professor Sashikumar Ganapathy, Senior Nurse Manager Lim Sok Lian, Nurse Clinician Zhang Rui Li, registered nurse (RN) Gladys Ng, RN Kai Lee Shih, RN Kathryn K. Tan, RN Minnie M. Tan, and RN Peggy Ang), as well as the participating patients and their families. In addition, the authors appreciate Quality, Safety and Risk Management team members Jeslyn Neo and Bernard Wong for providing QI methodology advice, data extraction, and statistical support.

## Supplementary Material


