# Supplementary figures and images for: Parent-administered Metered-dose Inhalers Improves Medication Administration Time in the Children’s Emergency
Source: Pediatr Qual Saf. 2026 Jul 20;11(4):e889. doi: 10.1097/pq9.0000000000000889 (PMC13375059; doi:10.1097/pq9.0000000000000889)

Supplementary Digital Content 1. Fishbone diagram

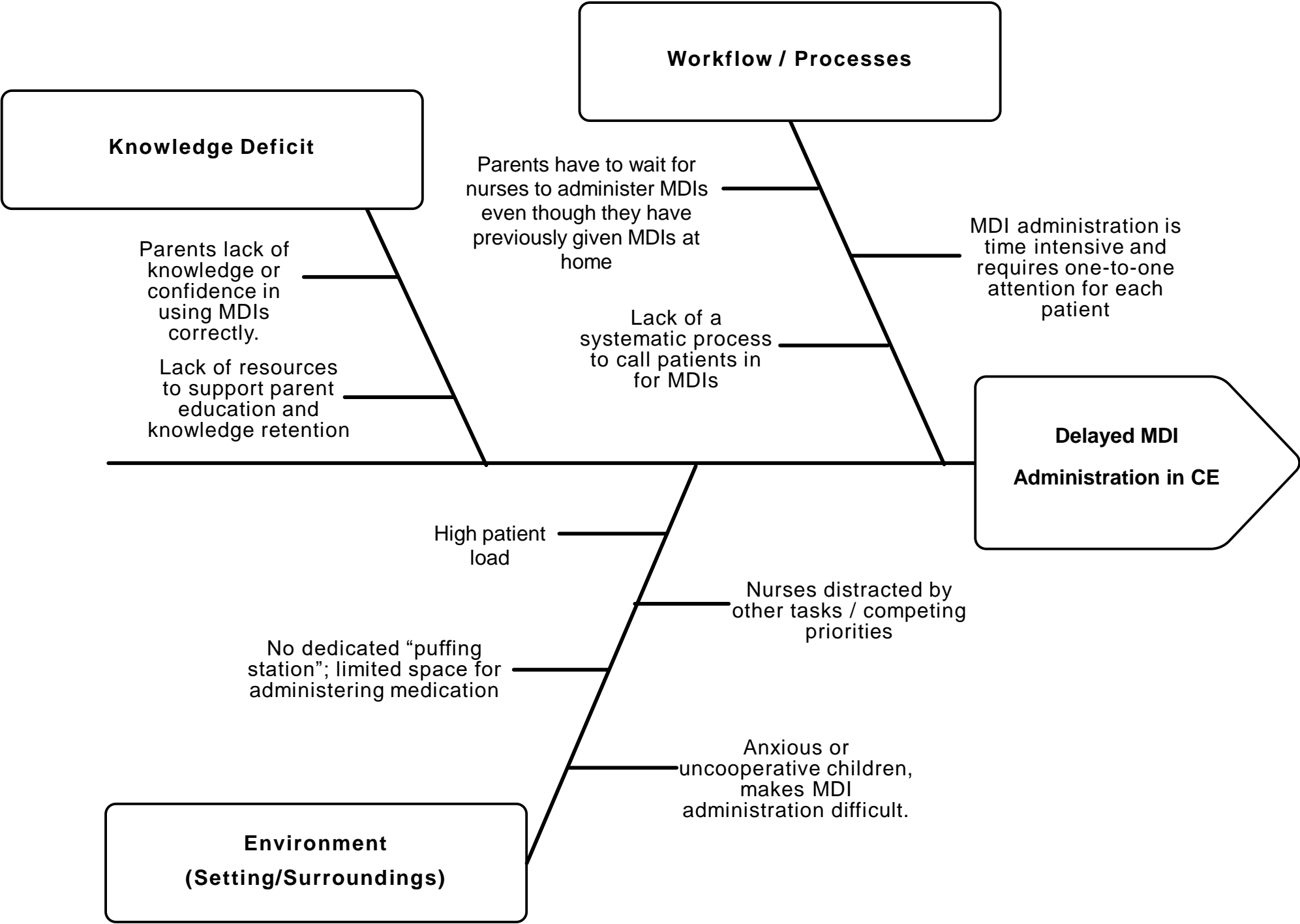

Supplement: Supplementary file 1 [file pqs-11-e889-s001.pdf]
