# Supplementary material for: Parent-administered Metered-dose Inhalers Improves Medication Administration Time in the Children’s Emergency
Source: Pediatr Qual Saf. 2026 Jul 20;11(4):e889. doi: 10.1097/pq9.0000000000000889 (PMC13375059; doi:10.1097/pq9.0000000000000889)
Supplement: Supplementary file 2 [file pqs-11-e889-s002.pdf]

Supplementary Digital Content 2. The Singapore Pediatric Triage Scale (SPTS)

**Triage Level/ Definition**	**Presentation**
Category 1/ Resuscitation (R)	• Respiratory/ cardiac arrest<br>• Grossly unstable vital signs/ Glasgow Coma Scale (GCS)<br>• Evidence of moderate-to-severe respiratory distress<br>• Signs of hypoperfusion (shock)<br>• Sepsis syndrome<br>• Severe dehydration<br>• Present seizure<br>• Altered mental state<br>• Febrile neutropenia (oncology patients with fever)<br>• Major trauma<br>• Anaphylaxis
Category 2+/ Non-Resuscitation (NR)<br>('9' case)	• Mild-to-moderate respiratory distress<br>• Post-fit, conscious<br>• Stable poisonings<br>• Moderate-to-severe pain<br>• Moderate dehydration (vital signs stable)<br>• Crush injuries<br>• Hyperpyrexia in children/ neonatal pyrexia<br>• Deformed fracture/ dislocated shoulder, elbow, patella or ankle<br>• Burns/ scalds 5%-9%<br>• Dislocated/ dislodged permanent tooth
Category 2/ Non-Resuscitation (NR)	• Minor trauma requiring procedures<br>• All afebrile infants < 3 months old (not having any P1/ P2+ criteria) and other indications<br>• Severity Index Score (SIS) 8 with acute complaints (e.g. diarrhea, abdominal pain with stable vital signs and reasonable hydration status)
Category 3/ Emergency	• SIS 9 and 10 with acute complaints (otherwise stable vital and normal activity level)

(From Source: Ganapathy S, Yeo J, Thia X, Hei G, Tham L. The Singapore Paediatric Triage Scale Validation Study. *Singapore Med J.* 2018;59(4):205-209. doi:10.11622/smedj.2017093)
