# Supplementary material for: Parent-administered Metered-dose Inhalers Improves Medication Administration Time in the Children’s Emergency
Source: Pediatr Qual Saf. 2026 Jul 20;11(4):e889. doi: 10.1097/pq9.0000000000000889 (PMC13375059; doi:10.1097/pq9.0000000000000889)
Supplement: Supplementary file 3 [file pqs-11-e889-s003.pdf]

KK WOMEN'S AND CHILDREN'S HOSPITAL
COMPETENCY CHECKLIST FOR PARENT– ADMINISTERED METERED DOSE INHALER (MDI) TECHNIQUE

Patient study ID: ___________________________

Date & Time: ___________________________

Instructions for assessor:

1. Assess each parents according to the performance criteria
2. Place a tick (√) for "Met" and a cross (X) for "Not Met" in the column provided for each competency
3. Provide feedback to parents after every MDI administration

S/N	CRITERIA	COMPETENCY ASSESSMENT		
		1	2	3
1.	Requisites preparation:<br>● Space chamber<br>● Appropriate mask size (infant or child mask)			
2.	Check and confirm the number of puffs to be given with the nurse<br>(Eg: Ventolin: __ puffs, Atrovent: __ puffs)			
3.	Remove cap from mouthpiece of inhaler			

4.	Shake inhaler before inserting into the space chamber			
5.	Insert inhaler in space chamber			
6.	Place appropriate mask size to the spacer over the mouth and nose			
7.	Ensure a tight fit of mask enclosing both nose and mouth firmly			
8.	Spray inhaler firmly pressing down the tip of canister once			
9.	Hold mask firmly to the face for 6 breaths			
10.	For another dose, wait 1 minute and repeat step 4 to step 9			
11.	Return the space chamber to the supervising nurse			

Competency Assessment 1

Competent ☐ Yes ☐ No Tick (√) accordingly

Comments:

Supplemental Digital Content 3. MDI competency checklist

Designation & Name of Assessor: ______________________

Signature of Assessor: ______________________

Date & Time: ______________________

Competency Assessment 2

Competent ☐ Yes ☐ No Tick (√) accordingly

Comments:

Designation & Name of Assessor: ______________________

Signature of Assessor: ______________________

Date & Time: ______________________

Competency Assessment 3

Supplemental Digital Content 3. MDI competency checklist

Competent ☐ Yes ☐ No Tick (√) accordingly

Comments:

Designation & Name of Assessor: ______________________

Signature of Assessor: ______________________

Date & Time: ______________________

(June 2022)
